# AI-Driven Rehabilitation Robotics: Advancements in and Impacts on Patient Recovery

**DOI:** 10.7759/cureus.94273

**Published:** 2025-10-10

**Authors:** Ghulam Husain Abbas, Caroline Speksnijder, Dharmanand Ramnarain, Chetan Parmar, Aum Parmar, Suhaib Ahmad, Sjaak Pouwels

**Affiliations:** 1 Faculty of Medicine, Ala-Too International University, Bishkek, KGZ; 2 Oral and Maxillofacial Surgery &amp; Special Dental Care, University Medical Center, Utrecht, NLD; 3 Intensive Care Medicine, Elisabeth-Tweesteden Hospital, Tilburg, NLD; 4 Surgery, Whittington Health NHS Trust, London, GBR; 5 Sciences, Sandringham School, St Albans, GBR; 6 General Surgery, Ysbyty Gwynedd Hospital, Bangor, GBR

**Keywords:** ai and machine learning, artificial intelligence, artificial intelligence and machine learning, assistive technology, intelligent prostheses, machine learning, prosthetics, rehabilitation robotics, robotics for rehabilitation, therapeutic robotics

## Abstract

The integration of artificial intelligence (AI) in rehabilitation robotics (RR) can be regarded as a significant advancement in patient recovery processes. RR refers to robotic technologies that are designed to support therapy and/or healing processes by applying tailored, adaptive, and efficient machine learning algorithms. This paper is a methodological narrative review discussing the latest trends in the application of AI in RR and its effects on the recovery of patients with stroke, spinal cord injury, and musculoskeletal disorders. Technological advancements in real-time monitoring, feedback, and intelligent data analysis have enhanced the effectiveness of these systems, providing patients with personalized rehabilitation programs that are progressive in nature, based on the patients’ improvement. Research evidence demonstrates that the use of AI-driven robotic systems in clinical practice significantly improves motor skills, the rate of recovery, and overall patients’ well-being, in contrast to conventional rehabilitation procedures. Moreover, the review highlights the drawbacks and problems that present-day technologies have; these problems concern the costs, the absence of unified protocols, and legal requirements. Furthermore, the combination of AI with RR will likely progress even more, with ongoing research studying ways in which the system’s intelligence could be upgraded, patients’ involvement in the process could be increased, and the applicability of these technologies to various forms of needs could be increased as well. The present review thus highlights the importance and the role of AI-RR in enhancing the patient’s recovery and opens the way for therapeutic practices. This narrative review was informed by targeted searches in PubMed, Scopus, and IEEE Xplore covering 2015-2025, with an emphasis on English-language, peer‑reviewed studies in stroke, spinal cord injury, and musculoskeletal rehabilitation; no formal meta-analytic synthesis was undertaken, consistent with a narrative approach.

## Introduction and background

Rehabilitation robotics (RR) is one of the promising subfields of health technology that holds the dreams and opportunities of millions of patients longing for a better life and a chance to be normal after critical health conditions [1]. This fairly new discipline lies in the middle of engineering and medicine and aims to use robotic tools to support and improve the healing process. Currently, the application of artificial intelligence (AI) in RR is in the process of a radical change, which will make the technology even more accurate, flexible, and efficient in terms of treatment [1]. Ranging from helping stroke survivors to assisting those with mobility issues, the goal of AI-assisted RR is not only to restore but also to additionally revolutionize lives. This review focuses on the important aspects of RR, starting with the concepts and the revolutionary application of AI [1].

Despite numerous reviews discussing the role of AI in RR, a clear gap remains in analyzing the evolving intersection of human-machine interaction, personalized therapy, and recent AI breakthroughs. This review distinguishes itself by providing a novel perspective that synthesizes advancements in AI-driven rehabilitation robots and their real-world impact on clinical outcomes. Additionally, it explores the practical challenges in implementation and ethical considerations, which have not been comprehensively addressed in prior works.

The objective of this review is to narratively synthesize recent evidence on AI-enhanced RR across neurological, musculoskeletal, and prosthetic domains, and to highlight opportunities, challenges, and future directions.

It goes further in looking into the analysis of how AI is changing, and how robotic systems carry out physiotherapy, from the use of exoskeletons in RR for tremor elimination to robotic rehabilitation therapy for stroke victims [1]. Furthermore, this review also wades through the progress made in the creation of prosthetics complemented by AI and supported by case studies that show actual achievements in the said field [1].

RR is composed of two major categories of robots: assistive and therapeutic. Assistive robots are designed to help individuals with disabilities perform daily tasks and enhance their independence. These robots provide physical support or compensatory assistance to users who have lost some functional ability due to injury, illness, or aging [2]. Therapeutic robots are designed to aid in the rehabilitation process by assisting patients in performing exercises and tasks that promote the recovery of lost functions. These robots are typically used in clinical settings under the supervision of healthcare professionals. Perfect examples of such are the Manus Assistive Robotic Manipulator, an assistive robot that replaces the missing limbs’ functions; it can be incorporated into devices like a powered wheelchair [1-3]. These robots are operated through different kinds of interfaces, and what is obtained is teleoperation, which is like the astronauts operating spacecraft arms. While there are social robots like PARO made by AIST, Japan, and KASPAR developed by the University of Hertfordshire in England, there are therapy robots like MIT-Manus founded by the Massachusetts Institute of Technology, USA, and Lokomat developed by Hocoma to practice and improve movement [1-3]. These robots help patients to execute individual movements that enhance their movement and use of the affected limbs necessary for those with spinal cord injuries, stroke, or musculoskeletal conditions. However, the prospects of the theoretical frameworks and approaches should not overshadow the difficulties and drawbacks that are present [3].

Finally, the review provides directions for future developments in AI and RR and presents the vision of the future where the synergy of technology and medicine offers patients needing rehabilitation truly unique prospects for improvement [3]. It will also analyze the technologies that have made these developments possible, the clinical results achieved from their application, and the possible prospects for further development of this branch of knowledge [3]. This review aims to discuss the role and opportunities of AI in RR and demonstrate how these innovations can enrich the treatment process and enhance patient efficiency.

## Review

Understanding RR

The development of RR was done at the end of the 20th century, targeting mostly patients with severe mobility difficulties. The first concepts were simple, mainly targeting the gross motor skills, and mostly needed a lot of help from therapists. Nevertheless, such features as sensors, actuators, and control algorithms that became integrated into these systems over the years have improved the characteristics of these systems step by step [4]. Just like in other medical specialties, RR is a major innovation aimed at helping people who have had a stroke, a certain injury, or who are faced with the problems of aging. This field combines the engineering aspect with health care to design and create robotic systems that assist in the rehabilitation process, and greatly improve patient outcomes and the ability to regain mobility [4]. Over the last few years, there has been a general enhancement of the effectiveness and availability of rehabilitation robots. Technologies that include AI, machine learning (ML), and real-time data processing have made such systems flexible in their approach toward patients, providing the best rehabilitation regimes that improve results.

However, with the advances of material science and portable designs, these devices are becoming increasingly portable and easy to use, thereby increasing their utilization in home environments and outpatient facilities [5]. The progress of the RR has benefited a lot from the neuroscience advancements that have established the neuroplasticity of the brain and spinal cord even under severe injuries [6]. Robotic systems in the initial years were programmed to help in executing simple functions, but the robots of the present day are far more advanced and are capable of performing complex operations, such as responding to voice or recognition of objects, and most of all, improvement from simple preprogrammed motor functions to obeying verbal commands as per patient needs [7].

This evolution is characterized by the use of AI and ML to make the robots effective in dealing with users and the surrounding environment. In addition, the field has witnessed a significant augmentation in the kinds of technologies applied to it [8]. Whereas early models of assistive technology were generally conceptualized as supporting simple limb movements, today’s systems comprise robotic exoskeletons and sophisticated orthotics [8]. Some of these improvements assist in the movement of the limbs and also aid in the rebuilding of the neural networks, which are helpful when treating neurological illnesses. The evolution of RR from simple assistive tools to sophisticated therapeutic systems showcases the growth and the future of the field as a potential game changer in medical practices and patients’ lives. Further development of these technologies remains highly potential to reshape the market of rehabilitative care and provide better opportunities for recovery to patients all over the world [8].

Methodology

This review employed a structured search strategy to identify relevant literature on AI in RR. Targeted database searches were conducted in PubMed, Scopus, and IEEE Xplore, covering the period 2015-2025, supplemented by manual screening of reference lists. Search queries combined MeSH terms such as “Artificial Intelligence,” “Machine Learning,” “Neural Networks,” “Rehabilitation Robotics,” “Exoskeleton Device,” “Prostheses and Implants,” “Stroke Rehabilitation,” “Spinal Cord Injuries,” and “Musculoskeletal Diseases,” with free-text keywords including “intelligent prosthetics,” “tele-rehabilitation robotics,” and “human-machine interface.” Studies were included if they were peer-reviewed, published in English, and investigated AI-powered or AI-assisted robotic systems for rehabilitation in patients with stroke, spinal cord injury, or musculoskeletal conditions, reporting outcomes such as motor recovery, gait improvement, functional independence, or patient engagement. Excluded were non-AI robotic studies, preclinical or animal research, simulation-only studies without clinical relevance, non-English publications, and articles focused on industrial or surgical robotics. Data extraction emphasized the type of AI systems, robotic systems, clinical applications, reported outcomes, and challenges. Consistent with a narrative methodology, no formal meta-analysis or risk-of-bias assessment was undertaken, but priority was given to high-impact, clinically relevant studies.

The integration of AI in RR

The use of AI in RR is an innovation in the field of medicine, especially in children and youth with disability or diagnosed health conditions. In this area, the most common AI approaches are robotics, which make up 77% of the studies, Human-Machine Interaction (HMI), Virtual Reality (VR), Natural Language Processing (NLP), ML, and Computer Vision [9-12]. Altogether, these technologies help to create better understandable and sensitive rehabilitation systems that can enhance patients’ outcomes. Analyzing AI-aided RR research, the emphasis has been made on the system of use, with 88% of robotics papers published in this area [12-14]. Such systems are intended to support sophisticated communications between patients and robotic tools with the goal of optimizing the therapeutic processes by increasing the flexibility and sensitivity of the response. Several studies reported statistically significant improvements in functional outcomes compared with conventional therapy (p < 0.05), with effect sizes ranging from small to moderate depending on the population studied [4-14].

HMI is vital in RR as 56% of the studies investigated use this technology [14-17]. It enables the patients to have better and efficient interaction with the robotic systems, and thus, the treatments being offered are more natural. For example, the Lokomat robotic gait trainer utilizes real-time adaptive algorithms to adjust robotic assistance based on patient performance, significantly improving motor relearning in stroke survivors. Similarly, the ArmeoSpring exoskeleton facilitates upper limb rehabilitation by incorporating biofeedback mechanisms, engaging patients in interactive exercises to promote neuromuscular recovery.

In 20% of cases, AI-driven visualization and VR are used, which creates more realistic conditions for patients’ training and also in home settings [18-21]. This technology is most useful in setting up artificial environments in which the patient can rehearse the various movements and activities, and it is also useful in both physical and cognitive recovery.

Both NLP and ML are essential for building better and smarter rehabilitation systems that can effectively cater to the patient’s needs more efficiently. These technologies are utilized in 19% and 12% of the studies, respectively, thus enhancing the systems’ capacity to accommodate patient-specific needs by leveraging big data analysis and ML [22-24].

CV is applied in 11% of the works and plays a crucial role in improving the interactivity of rehabilitation robots [25]. This technology allows robots to recognize and interpret the surrounding reality and has a positive effect on patient care and communication with them, particularly in cases when a patient needs assistance in certain complicated cases. AI is one of the fundamentals in the construction of an exoskeleton since it helps in numerous operations such as robot control, locomotion classification, detection, and human joints trajectory prediction [26].

Several prior studies have investigated diverse applications and methodological advances in AI-driven rehabilitation robotics, including adaptive assistance and control strategies, personalized gait-training algorithms, sensor fusion and intention detection, human-robot interaction frameworks, and predictive outcome modeling, which together provide the broader context for recent work in this area [27-39]. A 2023 study by Khalid et al. demonstrated that AI-driven robotic exoskeletons integrating gait pattern recognition could reduce rehabilitation time for post-stroke patients by 30% compared to conventional therapies [40].

These functionalities underline the individual approach to the treatment process and ensure that the exoskeletons are capable of responding to the wearer’s motions, thus improving the efficiency of the rehabilitation procedure [27]. Due to the integration of AI, the monitoring features of rehabilitation robots are improved. Such data collection is continuous, thus enabling assessment of the performance and progress of the patients. This data can be made available to the healthcare providers with detailed information about the patient’s rehabilitation process and help in decision-making in clinical practice [28].

Furthermore, the robots’ real-time feedback assists patients in correcting the movements during the course of physiotherapy, resulting in safer and more efficient exercises. The application of these AI technologies to RR not only improves the performance of the systems but also creates new opportunities for patient management; for example, AI facilitates remote monitoring, automated progress tracking, and more engaging, motivating therapy experiences, revolutionizing patient management and making rehabilitation more holistic and continuous, and thus improving the nature of rehabilitation.

AI-powered robotic systems for physiotherapy

Robotic systems that are enhanced by AI have greatly impacted the area of physiotherapy by providing state-of-the-art technologies to be incorporated in the care of patients. Therapeutic robots with the help of AI and depth cameras are engaged in the observation of the patient’s posture during exercises. These robots quantify the motion graduations and track the movements with a level of accuracy above human abilities. Also, such systems communicate with patients; apart from coaching, they offer encouragement, which is critical for patients’ morale and compliance with the prescribed rehabilitation schedule [29]. Social robots also have an important function in this domain. They interact with the patients through communication and motivation, which can go a long way in improving the rehab process. These interactions are useful in encouraging the patient’s compliance in the therapy sessions. AI integration in robotics provides numerous advantages for patients and their rehabilitation process, increasing the productivity of therapy sessions and adapting to patients’ needs [30].

AI technologies help physiotherapists to get the best results because they take into account factors such as age, gender, and medical history of the patient. Such an approach is useful in ensuring that treatments for individual patients are more efficient and responsive to the patient’s rehabilitation requirements. AI plays a significant role in determining a patient’s medical history and their current physical condition, thus assisting in the formulation of the right diagnosis as well as making adjustments to treatment options in real-time [31]. Such flexibility facilitates the delivery of care that is most appropriate to the stage and overall health of every patient (Table 1).

**Table 1:** Comparative Table for AI-Powered Systems AI, artificial intelligence.

System Type	Example	Key Features	Advantages	Limitations
Exoskeletons	Lokomat	Robotic-assisted gait training	Improves mobility in stroke and spinal cord injury patients	High cost, limited accessibility
Prosthetics	Utah Bionic Leg	AI-driven intent detection	Personalized movement adaptation	Requires extensive calibration
Virtual Reality (VR) Systems	MindMotion GO	Interactive neurorehabilitation games	Enhances patient engagement	Effectiveness varies by patient

Furthermore, AI-integrated movement monitoring systems give real-time feedback and comprehensive analysis during the therapy session. These systems employ sensors to capture and track detailed information on patients’ mobility, information that enables therapists to enhance the rehabilitation exercises. These systems are intended to overcome the shortcomings of conventional physiotherapy by delivering constant, precise, and versatile assistance based on the patient’s requirements. AI algorithms analyze vast amounts of data collected from sensors embedded in robotic devices, enabling real-time adjustments to therapy protocols based on patient performance and progress [32]. This data-driven approach ensures that exercises are optimally challenging and beneficial, promoting quicker and more comprehensive recovery. For example, in stroke rehabilitation, AI-powered exoskeletons can adapt to the varying degrees of motor impairment in patients, providing the necessary assistance or resistance to facilitate motor learning and neural plasticity. Similarly, for patients with spinal cord injuries, these systems can aid in gait training by dynamically adjusting the level of support provided during walking exercises, thereby enhancing mobility and reducing the risk of secondary complications such as muscle atrophy or pressure sores [33]. Through gamification elements and interactive feedback, patients are encouraged to actively participate in their rehabilitation, leading to better adherence to therapy regimes.

Furthermore, AI can analyze patient feedback and adjust the therapy to make it more enjoyable and less monotonous. These systems are also equipped with advanced computer vision technologies that enable precise monitoring of patient movements. This capability is crucial for ensuring exercises are performed correctly, minimizing the risk of injury, and maximizing therapeutic benefits. Real-time visual and haptic feedback provided by the robotic systems helps patients correct their form and movements, leading to more effective rehabilitation sessions. Another significant advantage of AI-powered robotic systems is their ability to facilitate remote therapy.

Tele-rehabilitation platforms integrated with AI and robotic systems enable patients to receive high-quality physiotherapy in the comfort of their homes. This is particularly beneficial for patients in remote or underserved areas who may not have easy access to specialized rehabilitation centers. Remote monitoring and data analysis allow therapists to track patient progress, adjust therapy protocols as needed, and provide timely interventions, ensuring continuity of care [34].

The implementation of AI in robotic physiotherapy systems also extends to predictive analytics. By analyzing historical data and patient-specific information, AI can predict potential setbacks or complications in the rehabilitation process and proactively adjust therapy protocols to mitigate these risks. This predictive capability enhances the overall efficacy of the rehabilitation program, leading to better long-term outcomes. However, the integration of AI-powered robotic systems in physiotherapy is not without challenges. Ensuring data privacy and security is paramount, as these systems handle sensitive patient information. Additionally, there is a need for standardized protocols and regulatory frameworks to ensure the safety and effectiveness of these advanced therapeutic tools. The high cost of AI-powered robotic systems also poses a barrier to widespread adoption, necessitating efforts to make these technologies more affordable and accessible. Looking forward, ongoing research and development are expected to further enhance the capabilities of AI-powered robotic systems for physiotherapy. Innovations such as more sophisticated AI algorithms, improved sensor technologies, and enhanced user interfaces will continue to drive the evolution of these systems [35].

Moreover, interdisciplinary collaboration among engineers, clinicians, and researchers will be crucial in addressing the existing challenges and unlocking the full potential of AI in robotic physiotherapy. These systems hold the promise of improving patient outcomes, increasing access to high-quality rehabilitation, and ultimately enhancing the quality of life for individuals undergoing physiotherapy. As technology continues to advance, the role of AI in robotic physiotherapy is poised to become even more significant, paving the way for innovative and impactful therapeutic solutions [36]. Furthermore, the ability of robotic systems to deliver therapy to multiple patients simultaneously without compromising the quality of care is a significant advancement. This addresses the high demand in clinics effectively, ensuring that more patients can receive timely and quality therapy. The precision of robotic systems also minimizes human error, providing consistent and reliable therapy sessions that contribute to quicker and smoother patient recovery.

AI in prosthetic development

AI is revolutionizing the field of prosthetic development, enhancing the integration and functionality of artificial limbs. Innovations in AI-assisted prosthetics are not only about data gathering and analysis but also about creating systems that learn and adapt over time. These advances promise a future where prosthetic devices can integrate more seamlessly with human physiology, adjusting as a person's habits and physical conditions change. Myoelectric prostheses equipped with AI can analyze muscle contractions and translate these signals into movements of the artificial limb, enabling users to perform complex tasks such as grasping objects with varying degrees of force or precision [37]. Similarly, AI algorithms integrated with pattern recognition technology can differentiate between different types of muscle signals, allowing for more accurate and varied control of the prosthetic limb. Moreover, AI is instrumental in enhancing the customization of prosthetics.

By leveraging ML, prosthetic systems can be tailored to individual users based on their specific needs, preferences, and physiological characteristics. This personalized approach improves comfort, functionality, and overall user satisfaction. For example, AI-driven adaptive algorithms can analyze data from a user’s gait and adjust the prosthetic’s movement patterns to better align with natural walking dynamics, thereby reducing discomfort and increasing efficiency.

The development of AI-enhanced prosthetics also includes the integration of advanced materials and design techniques. AI aids in the optimization of prosthetic designs by simulating various scenarios and materials to identify the most effective configurations. This process accelerates the development cycle and leads to the creation of lighter, more durable, and ergonomically designed prosthetic limbs. Furthermore, AI contributes to the field of bionic prosthetics, where advanced neural interfaces enable direct brain control of prosthetic devices. By using AI to interpret neural signals, these prosthetics offer a high degree of precision and functionality, allowing users to perform intricate movements such as typing or playing musical instruments.

Despite the significant advancements, the incorporation of AI in prosthetic development also presents challenges. Ensuring the reliability and safety of AI algorithms is critical, as any errors or inaccuracies in signal interpretation could impact the user’s performance or safety.

Smart prosthetics represent a significant leap forward in prosthetic technology. These devices incorporate advanced sensors and AI-driven control systems that enable real-time feedback and personalized adjustments. For instance, the development of prosthetic legs at North Carolina State University showcases how AI uses computer vision and other sensors to maintain a natural gait by adapting to different surfaces. Similarly, "smart skin" technologies for upper-limb prosthetics are equipped with touch-sensitive capabilities and onboard learning systems. These innovations allow the prosthetic to react appropriately to stimuli, with some models featuring multilayered sensors in artificial fingertips that detect force, temperature, and moisture, converting these into electrical signals for more intuitive use. Another notable advancement is in smart sockets, which utilize sensors to detect changes in the residual limb's volume over time, adjusting the socket for a secure and comfortable fit. These devices exemplify how AI can personalize user experience by learning individual preferences for fit and adapting to behavioral patterns. The integration of AI in prosthetic development significantly impacts patient mobility, particularly through the enhancement of lower-limb prosthetics. The Utah Bionic Leg is an example where intent detection is built into the prosthesis, going beyond traditional microprocessor knees. This system includes additional sensors that interpret signals from the residual limb muscles, facilitating more natural and intuitive movements. Moreover, some prosthetic limbs are now capable of tapping directly into peripheral nerves, with prototypes like those developed at the University of Minnesota [38,39]. These limbs use nerve implants to interpret brain signals, allowing fine motor control over individual fingers. Over time, the AI in these devices learns to correlate specific nerve signals with intended gestures, enhancing the user's control and independence.

Recent studies highlight the use of reinforcement learning in robotic prosthetics, where AI continuously refines movement precision through real-time feedback, enabling smoother and more natural motions. Ongoing research is focused on improving the robustness of AI systems, enhancing user interfaces, and exploring innovative materials and designs to address these challenges; despite the significant advancements, the incorporation of AI in prosthetic development also presents challenges (Table 2). Ensuring the reliability and safety of AI algorithms is critical, as any errors or inaccuracies in signal interpretation could impact the user’s performance or safety. The advancements in AI-enhanced prosthetics not only improve the functionality and natural feel of artificial limbs but also empower individuals with disabilities to achieve greater mobility and independence [40-58]. These technological strides are pivotal in improving the quality of life for amputees and those reliant on prosthetic devices.

**Table 2:** AI systems, orthotics, and their applications in robotic rehabilitation AI, artificial intelligence.

Type	Description	Examples	Role in Rehabilitation	References
Orthotics				
Ankle-foot orthotics (AFO)	Braces that control the position and movement of the ankle and foot	Hinged AFO, rigid AFO, dynamic AFO	Stabilize the ankle, improve walking patterns, reduce risk of falls	Bashir et al. [55]
Spinal orthotics	Braces designed to support or correct the spine	Scoliosis brace, lumbar support brace	Correct spinal deformities, reduce pain, improve posture	Devlin [56]
Upper limb orthotics	Devices that support the arm, wrist, or hand	Wrist splints, elbow braces, shoulder supports	Support injured limbs, improve function, reduce pain	Latour [57]
AI systems				
Robotic process automation (RPA)	AI that automates repetitive tasks	Workflow automation, data entry automation	Streamline administrative tasks, ensure consistency in therapy delivery	Madakam et al. [58]

Moreover, it must be considered how different prostheses have the potential to interact and combine. In a situation where a patient has separate impairments, there are two potential approaches: a split or unified system.

In a split system, preexisting industry solutions are more easily implemented, therefore not requiring any new development or testing. However, this could add another layer of complexity for the user as they would have to keep track of more than one maintenance schedule and get comfortable with different software solutions, further widening the "technology divide" [41].

On the other hand, a unified system will roughly have the opposite effect. One overarching user interface would make adoption easier, while the reduced number of potential failure points makes upkeep smoother. In addition, due to these systems being less common, each one can be even more personalized to the user. An example of this solution is a brain-actuated smart wheelchair and robotic arm for transportation [42].

Another aspect that needs to be noted is the upgrade path. In the situation of a major innovation in one prosthetic field, implementing this into a more integrated system will be much more difficult and may require an entire replacement. Overall, unified systems will be more expensive but have a better user experience, and vice versa for the split systems [43].

Future directions in AI and RR

The trajectory of RR is set toward integrating more sophisticated AI technologies that enhance human-machine interaction and improve patient outcomes. One significant area of focus is the development of brain-computer interfaces (BCIs), which are poised to revolutionize how patients interact with rehabilitation devices [44]. These interfaces will likely rely heavily on ML algorithms to process and predict patterns from large datasets, enhancing the system's responsiveness and adaptability to individual patient needs.

Moreover, the field is exploring the potential of electromechanical interaction and intelligent control systems. These systems aim to refine the rehabilitation robot's ability to act as a therapist, providing targeted and efficient therapy tailored to the patient's specific rehabilitation requirements [44]. The integration of feature engineering and dimensionality reduction techniques will further enhance the data processing capabilities of these robots, enabling them to deliver more precise therapeutic interventions.

Looking ahead, the integration of AI in RR holds promises for more personalized and accessible healthcare solutions. Innovations in material science and sensor technology are expected to drive the development of more advanced robotic prosthetics and exoskeletons. These devices will not only mimic natural limb functionality more closely but will also adapt dynamically to changes in the user's physiological condition, thereby enhancing the effectiveness of the rehabilitation process.

Another exciting development is the use of robotic care equipment in clinical settings. These robots will assist healthcare professionals by taking over repetitive or physically demanding tasks, thus reducing the strain on human caregivers and increasing the efficiency of the rehabilitation process [42-51]. This shift is expected to improve the quality of care provided to patients and allow for more personalized attention where it is most needed.

While BCIs hold great promise for rehabilitation, challenges such as signal reliability, long-term neural integration, and regulatory approval remain. Experts predict that commercially viable BCI rehabilitation systems may become clinically accessible within the next 5-10 years, pending advancements in non-invasive neurotechnology and real-time AI processing.

Furthermore, the seamless integration of intelligent assist devices with sophisticated human-machine interfaces will ensure that these technologies complement rather than replace human efforts [52]. This integration is crucial for creating a natural and effective rehabilitation experience, where technology acts as an enabler rather than a replacement.

These advancements signify a future where RR not only restores function but also enhances the quality of life for patients, making recovery from injury or disability more achievable than ever before [53,54]. As research continues and technology evolves, the possibilities for innovation in this field appear boundless, promising a new era of enhanced mobility and independence for individuals across the globe. There is a prospect of the development of future branches, which combine AI, robotics, and human psychology.

Challenges and limitations

Despite the significant advancements in RR enhanced by AI, several challenges and limitations persist that could hinder the broader implementation and effectiveness of these technologies. One of the primary challenges is the high cost associated with developing and implementing AI-driven robotic systems. The advanced technology, including sophisticated sensors, ML algorithms, and real-time data processing capabilities, contributes to the significant expense of these systems. This cost can limit accessibility for patients and healthcare facilities, particularly in underserved or resource-limited settings. Moreover, the high cost of maintenance and calibration of these systems adds an additional financial burden, which can impact their long-term feasibility and widespread adoption. Another significant challenge is ensuring the reliability and safety of AI algorithms used in RR. AI systems depend on complex algorithms that must accurately interpret data from sensors and adjust robotic actions accordingly [41]. However, imperfections in these algorithms or errors in data interpretation can lead to incorrect therapeutic interventions, potentially causing harm or impeding recovery. Ensuring the robustness and accuracy of these algorithms through rigorous testing and validation is crucial, yet it remains a challenging and resource-intensive task.

Furthermore, the integration of AI in RR raises concerns about data privacy and security. These systems collect and process sensitive patient data, including physiological measurements and personal health information. Protecting this data from breaches and unauthorized access is essential to maintain patient confidentiality and trust. Developing and implementing robust data protection measures and complying with relevant regulations are necessary to address these concerns, but they add to the complexity and cost of these systems. The adaptability and personalization of AI-driven rehabilitation robots, while advantageous, also present challenges. These systems are designed to adjust therapy protocols based on real-time data and patient progress.

Additionally, the cost of AI-powered prosthetics remains a barrier to widespread adoption, necessitating efforts to make these technologies more affordable and accessible. However, achieving precise and effective personalization requires accurate and comprehensive data on each patient’s condition and response to therapy. Inadequate data or variability in patient responses can hinder the system’s ability to deliver optimal and individualized care. Additionally, designing AI systems that can effectively accommodate a wide range of patient needs and conditions is a complex task, necessitating ongoing research and development. User acceptance and engagement with AI-driven rehabilitation robots also pose challenges. Patients and healthcare providers may have varying levels of comfort and trust in using advanced robotic systems, particularly those driven by AI [42]. Overcoming skepticism and ensuring that users understand and feel confident in the benefits of these technologies is essential for successful implementation. This requires effective communication, education, and user training to facilitate the adoption of AI-powered rehabilitation tools. Additionally, the integration of AI in RR often involves interdisciplinary collaboration among engineers, clinicians, and researchers. Coordinating these diverse expertise areas and aligning their objectives can be challenging, requiring clear communication and collaboration to ensure that the systems developed meet clinical needs and are practical for real-world use. Regulatory and ethical considerations surrounding AI-driven RR need to be addressed. Establishing standards and guidelines for the development, testing, and deployment of these systems is essential to ensure their safety, efficacy, and ethical use. Regulatory frameworks must evolve to keep pace with technological advancements, providing clear and consistent guidelines for developers and users [43]. Addressing these challenges requires a concerted effort from stakeholders across the healthcare, technology, and regulatory sectors. Addressing these limitations through ongoing research, technological advancements, and collaborative efforts is crucial to unlock the full potential of AI in RR and ensure that these innovations can benefit a wide range of patients and healthcare providers.

One of the AI-based concerns in the integration of AI with RR is system reliability. The heterogeneous nature of data reporting across different sources can lead to biased, inaccurate, or inexecutable AI models [44,45]. This variability poses a significant challenge as it affects the interoperability of data essential for curating effective AI models. Moreover, the black-box nature of AI algorithms contributes to a lack of transparency, which can limit their adoption. The uncertainty about the computational processes, the validity of AI predictions, and inevitable model errors during initial deployment and ongoing tuning can generate distrust among healthcare providers. To address these issues, there is a need for standardized data reporting and the development of accountable, user-friendly guidelines that help interpret AI predictions and identify potential sources of error.

Various factors influence the perceptions and acceptance of AI among patients and healthcare professionals. For instance, there is a reported fear of loss of professional autonomy and difficulties in integrating AI into clinical workflows, which are unanimously seen as hindering factors. Additionally, the sensitivity of AI-driven alerts can lead to alarm fatigue, characterized by an increase in a clinician's response time and/or a decrease in response rate to clinical alarms due to too many alerts [46]. This phenomenon can compromise the safety of patient care, as overly sensitive alarms may induce desensitization and alert dismissal. Furthermore, technical reservations such as unintuitive user interfaces and limitations like the unavailability of strong internet connections impede the comprehensive usage and acceptance of AI [46].

User skepticism toward AI-driven RR stems from concerns about safety, reliability, and usability. To enhance trust and adoption, strategies such as transparent AI decision-making, user-friendly interfaces, and co-design approaches that involve both patients and healthcare providers are essential. Clinical validation studies demonstrating efficacy and safety will further improve acceptance among practitioners [46,47].

In addition to cost and technical concerns, ethical considerations such as patient safety, data privacy, and regulatory oversight remain central challenges for clinical adoption [47-52].

Addressing these challenges requires a multifaceted approach involving close collaboration between AI developers, healthcare providers, institutional regulators, and policymakers. Enhancing the transparency and actionability of AI insights, improving the interoperability of healthcare data, and ensuring the ethical use of AI are imperative to overcome these barriers and fully realize the potential of AI in RR [47-54].

## Conclusions

In conclusion, given the centrality of AI to the progress of RR, we have discussed how the incorporation of the two is revolutionary in the process of patient recovery and the general improvement of prosthetics. The improvements in accuracy, flexibility, and efficacy that are provided by AI-based systems have far-reaching implications for everyone in the process of rehabilitation, offering not only better odds of successful physiotherapy but also a major advancement in the capabilities of prosthetics. Through highlighting the aspects of a personalized approach to treatment, accurate assessment of the patient’s progress, and the creation of prosthetic limbs that are even closer to being natural, this review has outlined the direction toward the future of rehabilitation that is more efficient and accessible. The opportunities of RR are in the ability to use technologies to enrich patient care and be an example of AI’s benefits. These challenges and limitations lay the groundwork for further research and development initiatives, calling for more and improved work to overcome these barriers. This review, while narrative in scope, acknowledges methodological limitations and emphasizes ethical, technological, and clinical considerations that will shape the field going forward. Thus, the further enhancement of the integration of AI technologies into RR can be expected to continue this transformation process and result in new advancements that will create new benchmarks for patient care, increase patients’ independence, and improve the quality of life of the people, all in the process of rehabilitation.
